# Theoretical and Experimental Research Concerning the Friction Forces Developed in Hydraulic Cylinder Coaxial Sealing Systems Made from Polymers

**DOI:** 10.3390/polym16010157

**Published:** 2024-01-04

**Authors:** Flavius Aurelian Sârbu, Felix Arnăuţ, Andrea Deaconescu, Tudor Deaconescu

**Affiliations:** Department of Industrial Engineering and Management, Transilvania University of Brasov, 500036 Brasov, Romania; sflavius@unitbv.ro (F.A.S.); felix.arnaut@unitbv.ro (F.A.); deacon@unitbv.ro (A.D.)

**Keywords:** coaxial sealing systems, hydrodynamic friction, pressure distribution, PTFE, polyurethane

## Abstract

Optimizing the energy efficiency of hydraulic cylinder modern sealing systems requires, among other things, minimizing the developed friction forces. This can be achieved by manufacturing seals from polymer-based polytetrafluoroethylene-type materials (Virgin PTFE and filled PTFE) or from thermoplastic polyurethane elastomers. This paper presents a procedure for calculating and experimentally determining the friction forces developed in the coaxial sealing systems of hydraulic cylinders pistons. Three sealing systems made from different materials were tested under varying conditions of pressure and velocity on an experimental test stand set up by the authors. The paper concludes with data and recommendations for the selection of the optimum seal material in order to maximize energy efficiency. Our comparative research conducted on the seal materials led to the conclusion that for reducing friction forces in hydraulic cylinders, Virgin PTFE is the most adequate.

## 1. Introduction

Hydraulic actuation systems are used in industrial applications that require ensuring high forces, torques, rigidities, and durability in high-energy density systems. Regarding energy performance, however, hydraulic actuation systems have an efficiency of merely 21% [1]. This less than satisfactory characteristic calls for an optimized design/redesign of all hydraulic system components, and particularly of hydraulic motors.

The main causes for the energy loss that affects hydraulic actuation systems include the development of high friction forces and heat, as well as fluid leakages. Energy optimization in hydraulic cylinders mainly involves reducing the friction forces in the system. In addition to increasing energy efficiency, reducing the friction forces is also required for better mobility control of the hydraulic cylinder in terms of reproducibility and accuracy.

Reducing the friction forces in hydraulic cylinders entails the conception of optimized sealing systems based on reliable data concerning seal geometry and materials, the compatibility of the seal material and fluid(s) used, the dynamics of the sealing process, contacting surface roughness, etc. An optimum combination of all these elements yields increased operational efficiencies for hydraulic cylinders.

The sealing process of hydraulic cylinders is a complex procedure meant to ensure the complete separation of two different mediums: oil and air. Dynamic sealing is of particular interest for increasing energy efficiency. This type of sealing applies to cases when one of the sealed-off surfaces is more mobile in relation to the other. Concretely, this means sealing off the piston from the boring of the cylinder, or sealing off the cylinder rod from the cap of the linear hydraulic motor.

The dynamic sealing system of hydraulic cylinders consists of an assembly of elements, the principal one being the seal itself. A seal can be deformable or non-deformable, and it is placed in a specially designed seat. Due to its precompression at mounting and/or by means of the fluid pressure, the seal is pressed onto the sealed surface, thus ensuring the hermetic sealing of the system [2].

A performant sealing system has to meet two major requirements simultaneously: small friction forces and no fluid leakage. Intuitively reducing friction forces would entail less pressure being exerted by the seal onto the sealed surface. Such a course of action would, however, cause fluid leakage, thus compromising the very role of the entire sealing system. As, notably, these two requirements are contradictory, the situation can be solved by optimizing the design of the seal geometry and by selecting an adequate seal material that ensures small friction coefficients.

A dedicated redesign of sealing systems is also called for, considering that, as concluded by Oprean et al. [3], the sealing is responsible for 44% of the operational break-downs of hydraulic cylinders.

While, in the existing literature, numerous research studies can be found on the performance of hydraulic cylinder sealing systems, it needs be pointed out that these studies concern specific concrete sealing systems, and their results cannot be generalized. These studies were aimed at optimizing either seal geometry or the selection of adequate materials in order to reduce friction forces.

An example of a study that focused on seal geometry is that of Ma et al. [4], wherein the authors present a test rig and the results they obtained after testing two sealing systems of hydraulic cylinder piston rods (the so-called M-sealing system and T-sealing system, respectively). The authors concluded their paper by recommending the M-system for stable operations conditions, while the T-system lends itself to high-frequency working conditions.

Barillas et al., in [5], analyzed the performance of hydraulic cylinder sealing systems as assemblies, taking into consideration the actual sealing elements, the wipers, and the rod/piston bearing elements. After considering various geometries, materials, and working parameters (temperatures, pressures), the main conclusion of the study was that selecting any given sealing system should be based on a comprehensive analysis rather than an individual analysis of each component.

In [6], Nikas asserts that the operational efficiency of dynamic sealing systems depends largely on the geometry of a small gap that, in the literature, is referred to as the “elastohydrodynamic (EHD) inlet zone”, which is what the fluid is driven into during operation. In this zone, the pressure gradient is an important factor that determines the dynamic generation of thin lubricant layer. This layer separates, partially or integrally, the seal from the sealed surface. Depending on the thickness of this layer, which is typically smaller than 1 µm, the performance of the sealing system is described by the friction force and abrasive wear rate of the tribosystem [6].

Similar research regarding the formation of a fluid film at the seal and the sealed-off surface interface was carried out by Deaconescu et al. in [7]. In this study, a methodology for calculating the mean height of the fluid layer is presented and subsequently validated experimentally.

Cheng et al. studied [8] the influence of working conditions on the performance of an axial sealing system. In this study, finite element analysis was used to characterize the influence of fluid pressure and the precompression of the O-ring on the sealed space’s degree of hermetization.

Modern sealing systems of hydraulic cylinders are made mostly from polymers, particularly plastomers, elastomers, or thermoplastic elastomers. The optimum combination of the paired materials (i.e., seal and working fluid) is selected by taking into account their compatibility as well as operational specifics like temperature and pressure. A large number of published papers address the utilization of plastomers for performant sealing systems. Weber et al. [9] looked at limiting the wear of seals by optimizing the geometry of PTFE lip seals via numerical simulation. Sujuan et al. studied [10] the behavior of seals made from pure polytetrafluoroethylene (PTFE) or PTFE + bronze, glass fiber, carbon fiber, carbon, or graphite at environmental and high temperatures, respectively. The results confirmed that material mixing increases the wear resistance of the entire sealing assembly.

Polytetrafluoroethylene (PTFE) has a small friction coefficient (0.05–0.1), a most useful property in tribological applications conceived to reduce energy consumption in friction-intensive machinery. For this reason, this paper focuses on the study of sealing systems made from PTFE or adulterated PTFE. Another analyzed material is polyurethan, which, in combination with solid lubricants, yields small friction coefficients.

For the last few decades, hydraulic cylinders have been equipped predominantly with coaxial sealing systems made from friction materials with improved properties. Classical elastomer-type materials have been replaced by others based on plastomers, thermoplastic elastomers, or duromers known for their remarkable antifriction qualities. Within this context, this paper presents theoretical and experimental results concerning the improvement of the energy efficiency of hydraulic cylinder coaxial sealing systems. The parameter used to characterize and improve efficiency is the friction force, which has to be as small as possible. The materials we studied are Virgin PTFE, PTFE 25% Glass, and H-PU 55D (polyurethane).

Regarding this paper’s overall structure, the Introduction is followed by a second section that discusses the structure of a coaxial sealing system and the sealing mechanism. The distribution of the pressures developed between the seal and the sealed-off surface is analyzed, and the computational relationships for the developed friction forces are deduced and presented. The third section discusses the experimental results obtained by testing three coaxial sealing systems made from different polymer materials. Also discussed are the dependencies of the friction forces on piston velocity and fluid pressure. The last section includes the main conclusions that can be derived from the study.

## 2. Materials and Methods

The circular groove in the piston of the hydraulic cylinder first houses, in the radial direction, an O-ring and, on top of it, the actual ring-shaped seal, which is pressed against the inner cylinder sleeve by this O-ring. The seal material has to ensure good sliding.

**Figure 1:**
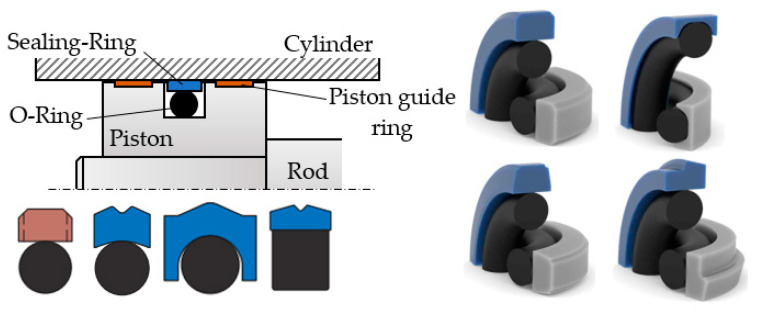
Coaxial sealing systems.

Figure 1 shows an example of a sealing system involving a piston and different cross-section geometries of the sealing ring [11,12].

Virgin or filled polytetrafluoroethylene, in short PTFE, are the seal materials favored in industrial applications. As is widely known and discussed in the literature, O-ring materials are elastomers; examples of elastomers include the following: hydrogenated nitrile butadiene rubber (HNBR), nitrile butadiene rubber (NBR), fluorosilicone (FVMQ), fluorocarbon (FKM), ethylene propylene diene monomer (EPDM), and silicone rubber (Q).

The circular groove in the piston is known as the O-ring’s seat, which ensures its preliminary radial compression of *ε_r_*_0_ = 10–25%. Once the piston is set into motion by the pressure exerted by the fluid, the deformation of the O-ring becomes greater and causes it to apply a larger radial force onto the sealing ring.

In a regime of hydrodynamic lubrication, the piston’s motion in the static cylinder boring causes the separation of the sealing ring from the sealed-off surface. This is due to the build up of a dynamic pressure and of a fluid layer of variable thickness in the initial contact area. Depending on the piston’s velocity *v* and on the dynamic viscosity *η* of the working fluid, the frictions conditions in the sealing tribosystem can be dry, fluid, or mixed. Regarding energy efficiency, fluid friction is preferable because of the significantly diminished friction forces. Fluid friction, however, requires a thicker fluid layer, which in its turn leads to leakage due to imperfect sealing. Thus, it follows that the presence of a fluid layer between the elements of the tribosystem offers the benefit of diminishing friction forces while carrying the inconvenience of leakage.

**Figure 2:**
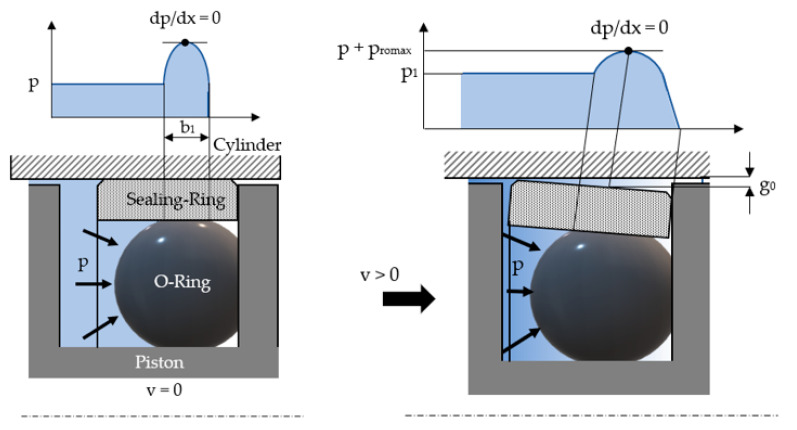
The formation of the fluid layer upon the onset of relative motion.

The type of friction in a coaxial sealing system is determined by calculating the average gap *g* between the sealing ring and the surface it comes into contact with. Figure 2 shows the process underlying the formation of this gap, along with a sealing ring of a rectangular cross-section.

The notations in Figure 2 can be explained as follows: *b*_1_ = the width of the contact surface between the O-ring and the sealing ring, *p_romax_* = the maximum contact pressure, and *g*_0_ = the thickness of the fluid layer in the point of the zero-pressure gradient (*dp/dx* = 0).

The sliding of the piston causes friction between the sealing ring and the inner sleeve of the cylinder. This friction is partially dry and partially hydrodynamic and is expressed by the respective forces *F_fr_d_* and *F_fr_HD_.* Equation (1) expresses the total friction force obtained by adding the dry and hydrodynamic components to each other.

(1) $$F_{fr}=F_{fr\_d}+F_{fr\_HD}=p\cdot \mu \cdot \beta \cdot A_{n}+\tau_{HD}\cdot (1-\beta )\cdot A_{n}$$

In Equation (1), *p* = the pressure of the working fluid; *μ* = the dry friction coefficient; *β = Ar/An* = the real non-dimensional contact area; *An*, *Ar* = the nominal and real contact area, respectively; and *τ_HD_* = the tangential hydrodynamic tension. The value of the real non-dimensional contact area *β* is less than unity and takes into account the characteristics of the sealing ring and cylinder materials, as well as the initial specific radial deformation of the O-ring *ε_r0_* and the pressure of the working fluid.

Equation (2), shown below, can be obtained by introducing the computational formula of the tangential hydrodynamic tension *τ_HD_* into Equation (1) [13].

(2) $$F_{fr}=F_{fr\_d}+F_{fr\_HD}=p\cdot \mu \cdot \beta \cdot A_{n}+2\cdot (1-\beta )\cdot A_{n}\cdot ln\left(\frac{1}{\beta }\right)\cdot \frac{\eta \cdot v}{g_{0}}$$

Further on, the flow of the fluid in the gap between the sealing ring and the inner surface of the cylinder is studied starting with the following hypotheses [7]:

- The preliminary compression of the O-ring in the static piston is greater than the compression of the seal, which has the same thickness as the fluid film.
- The pressure of the O-ring, which acts upon the seal, is distributed similarly to the fluid pressure distribution in the gap.
- In the radial direction, the hydrodynamic pressure in the gap is always compensated by the pressure resulting from the O-ring’s deformation.
- The pressure resulting from the O-ring’s deformation is distributed in a manner that defines the shape of the gap.

The magnitude of the gap *g*_0_ is determined based on the hypothesis that the sealing ring is a thin-walled cylinder subjected to internal pressure [7]:

(3) $$g_{0}=\sqrt[{3}]{\frac{3}{16}\cdot \frac{{\left(D-h\right)}^{2}}{E_{p}\cdot h}\cdot \eta \cdot v\cdot L\cdot (1-\beta^{2})\cdot \left[1-\frac{2\cdot \cosh(k\cdot L)\cdot \cos\left(k\cdot L\right)}{{cosh}^{2}\left(k\cdot L\right)+{cos}^{2}(k\cdot L)}\right]}$$

where

(4) $$k=\sqrt[{4}]{\frac{12\cdot (1-m_{p}^{2})}{h^{2}\cdot {\left(D-h\right)}^{2}}}$$

where *m_p_* and *E_p_* are Poisson’s ratio and the Young’s modulus of the seal material, respectively; *h* = the thickness of the seal; *D* = piston diameter.

The variation in the pressure in the gap can calculated using Equation (5), as deduced in [7]:

(5) $$p(x)=p+3\cdot \frac{\eta \cdot v\cdot L\cdot (1-\beta )}{g_{0}^{2}}\cdot \left[1-\frac{L\cdot (1-\beta )}{2\cdot x}\right]$$

where *p* = the pressure of the working fluid; *v* = the sliding velocity; *L* = the width of the seal.

**Figure 3:**
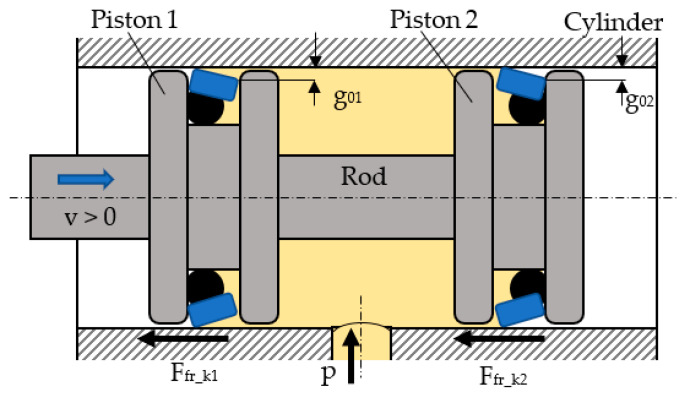
The construction of the tested hydraulic cylinder.

Figure 3 presents a simplified longitudinal section of the hydraulic cylinder that was further used for the experimental determination of the friction forces generated by the sealing rings.

The hydraulic cylinder has two identical pistons; hence, the coaxial sealing systems were tested pairwise. The fluid was introduced under pressure between the pistons into the mobile assembly that, initially, was at rest due to the fact that the axial forces caused by the fluid pressure were balanced against each other. In order to determine the friction forces generated by the two coaxial sealing systems, the pistons were set into motion by an exterior force that is not represented in Figure 3. The system consisting of the pistons and their seals moved against the kinematic friction in the coaxial seals. Each of the two tested sealing rings caused a kinematic friction force, $F_{fr\_k1}\;and\;F_{fr\_k2}$, respectively. The result of these two friction forces gives the total force $F_{fr\;k\;tot}$, which has to be overcome by the piston assembly.

(6) $$F_{fr\;k\;tot}=F_{fr\_k1}+F_{fr\_k2}$$

The friction forces generated by the two sealing rings are not equal. Their respective computational relationships were deduced in [13] and are as follows:

(7) $$F_{fr\_k1}=p\cdot \mu \cdot \beta \cdot A_{n}+2\cdot (1-\beta )\cdot A_{n}\cdot \frac{\eta \cdot v}{g_{01}}\cdot \ln\left(\frac{1}{\beta }\right)$$

(8) $$F_{fr\_k2}=p\cdot \mu \cdot \beta \cdot A_{n}+2\cdot (1-\beta )\cdot A_{n}\cdot \frac{\eta \cdot v}{g_{02}}\cdot \ln\left(\frac{1}{\beta }\right)$$

The system of Equation (9), consisting of Equations (6)–(8), is used to determine the value of each of the friction:

(9) $$\left\{\begin{array}{c} F_{fr\_k1}+F_{fr\_k2}=F_{fr\_tot}\\ F_{fr\_k1}-F_{fr\_k2}=2\cdot (1-\beta )\cdot \ln\left(\frac{1}{\beta }\right)\cdot A_{n}\cdot \eta \cdot v\cdot \left(\frac{1}{g_{01}}-\frac{1}{g_{02}}\right) \end{array}\right.$$

Adding the two equations above gives the following:

(10) $$F_{fr\_k1}=\frac{F_{fr\_tot}}{2}+(1-\beta )\cdot \ln\left(\frac{1}{\beta }\right)\cdot A_{n}\cdot \eta \cdot v\cdot \left(\frac{1}{g_{01}}-\frac{1}{g_{02}}\right)$$

Additionally,

(11) $$F_{fr\_k2}=F_{fr\_tot}-F_{fr\_k1}$$

The thicknesses of the fluid layers *g*_01_ and *g*_02_ are not equal because the hydrodynamic pressures that act upon the two sealing rings for any given direction are different. For the case presented in Figure 3, the equations of the two pressures are as follows:

(12) $$p_{1}=p+3\cdot \frac{\eta \cdot v\cdot L\cdot (1-\beta )}{g_{01}^{2}}\cdot \left[1-\frac{L\cdot (1-\beta )}{2\cdot x}\right]$$

(13) $$p_{2}=3\cdot \frac{\eta \cdot v\cdot L\cdot (1-\beta )}{g_{02}^{2}}\cdot \left[1-\frac{L\cdot (1-\beta )}{2\cdot x}\right]$$

The next section discusses the determination of the friction forces developed by the coaxial sealing systems made from different polymer materials based on the equations above and by means of an experimental test stand set up by the authors.

## 3. Experimental Results

The tests focused on three different sealing ring materials that are relevant in industrial applications: H-PU 55D, a TPU (thermoplastic polyurethane) that ensures auto-lubrication and two polytetrafluoroethylenes, namely PTFE 25% Glass and Virgin PTFE.

The three materials were selected for the following reasons:

- Their low friction coefficients for all the studied materials.
- They are the most frequently used materials for sealing systems.
- Polytetrafluoroethylene-type materials are resistant to almost all chemicals.
- Polyurethane elastomers are resistant to oil, petrol, hot water, hot air, and ozone.

In all tested systems, the O-rings were made from nitrile butadiene rubber (NBR) with a hardness of 70 Shore A. Table 1 presents the relevant characteristics of the materials used in our tests [14].

**Figure 4:**
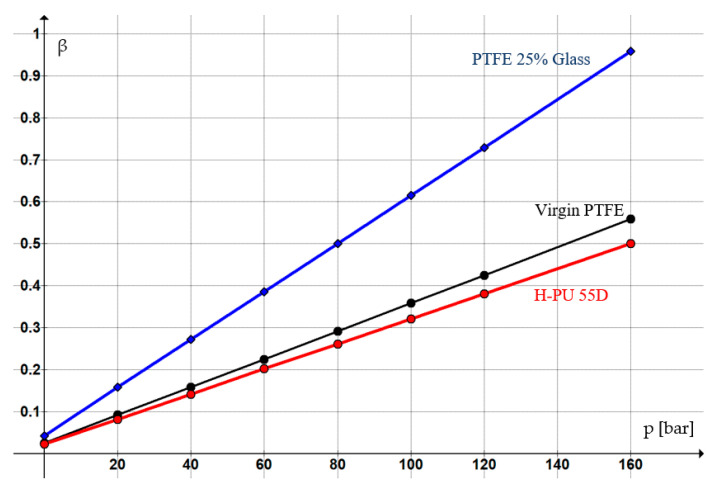
Dependency of the non-dimensional contact surface area on sealing ring material and pressure.

Table 2 shows the values of the non-dimensional contact surface area *β* for these materials [15], and Figure 4 presents, for each sealing material, the variation of *β* versus the sealed-off pressure.

It can be observed that as the pressure of the sealed-off fluid increases, the magnitude of coefficient *β* is greater, indicative of increased dry contact areas. For a given working pressure, coefficient *β* will be greater if the flow limits of the materials are smaller (σ _H-PU 55D_ > σ _Virgin PTFE_ > σ _PTFE 25% Glass_ → β _H-PU 55D_ < β _Virgin PTFE_ < β _PTFE 25% Glass_).

**Figure 5:**
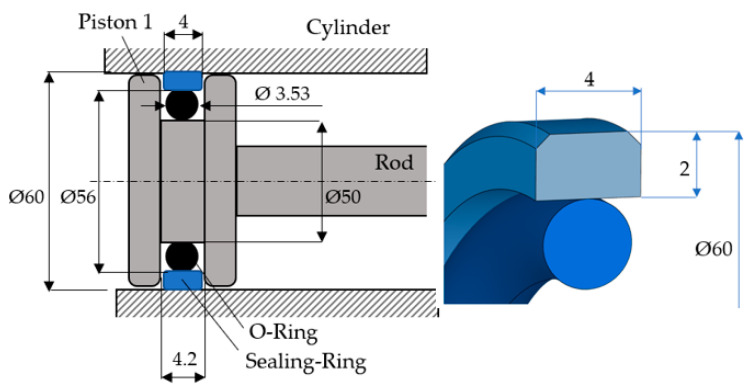
Dimensions of the coaxial sealing systems.

Figure 5 shows the dimensions of the tested coaxial sealing systems (all dimensions are in mm). The seals were machined by turning on a NC lathe; because of the low thermal conductivity of PTFE, the temperature must be carefully regulated while turning. This entails using very sharp tools and abundant colling liquid. The following cutting parameters were selected for turning Virgin PTFE: cutting speed—270 m/min; feed rate—0.08 mm/rev.

In this case, the initial specific radial deformation *ε_r_*_0_ is 15%. The material of the cylinder body that houses the two pistons is OLC45 steel (heat-treated quality carbon steel; DIN EN AISI 1.0503 C45-1045).

**Figure 6:**
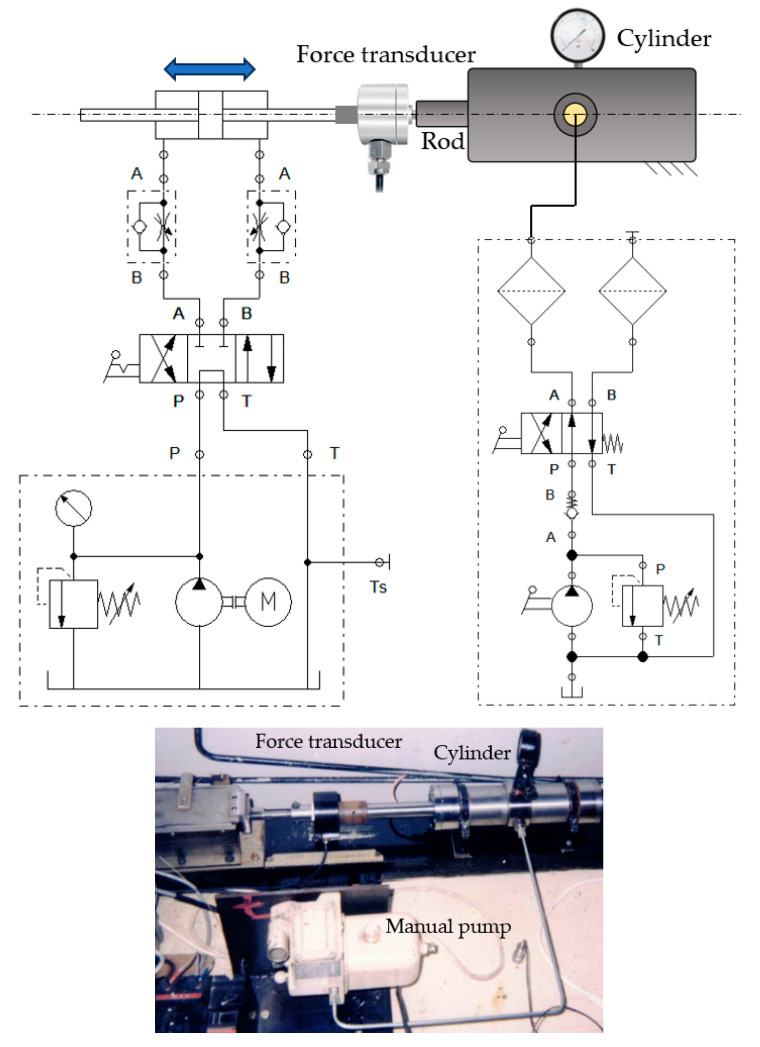
A diagram and an image of the test stand used for the determination of the friction forces.

Figure 6 shows a diagram and an image of the experimental test stand.

The cylinder used for testing the coaxial sealing systems is fed pressurized fluid by means of a manual pump that can ensure pressures up to 250 bar. The pistons can be moved into one or the other direction by means of a second hydraulic cylinder (on the left-hand side in Figure 6) that is fed by a constant flow pump and controlled by a 4/3 valve. The two hydraulic cylinders are connected via a force transducer. The working fluid we utilized was Anti-wear Hydraulic Oil ISO VG 32.

On this test stand, the friction forces caused by the contact between the sealing rings and the inner surface of the cylinder were measured by experimenting with the following material pairs: OLC45/Virgin PTFE, OLC45/PTFE 25% Glass, and OLC45/H-PU 55D, respectively. Furthermore, of importance for the measurements is the roughness of the contacting surfaces. Thus, the roughness of the cylinder surface is *Ra* = 0.2 µm, and the roughness values of the sealing rings are, for Virgin PTFE, *Ra* = 3.2 µm; for PTFE 25% Glass, *Ra* = 2.57 µm; and, for H-PU 55D, *Ra* = 3.2 µm.

For the OLC45/Virgin PTFE and OLC45/PTFE 25% Glass pair, the total friction forces developed by the two sealing systems were measured versus the velocity of the pistons and the working fluid pressure. The velocities were set at small values, at the limit between continuous and intermittent motion. These are the specific feed rates of machine-tools or the velocities of precise positioning systems actuated by hydraulic cylinders. At small velocities, the height of the fluid layer formed between the sealing ring and the cylinder is also small, leading to high friction forces. The bonding forces between the seal and cylinder materials that are in contact, as well as the respective roughness of the contacting surfaces, are responsible for the high values of the friction forces.

For each of the tested materials, five sets of friction force measurements were carried out. The arithmetic means and standard deviations were computed for the measured results. Table 3, Table 4 and Table 5 show the arithmetic means of the measured friction forces, as well as the standard deviation values.

Equations (10) and (11) were then used to determine the friction forces generated individually by each of the sealing systems.

In the case of the sealing rings made from H-PU 55D, intermittent gliding occurs in the analyzed range of velocities, which causes very large amplitude variations in the friction forces around average values. For this reason, Table 5 shows only average values calculated using Equation (14):

(14) $$F_{fr\_k\_med}=\frac{F_{fr\_tot}}{2}$$

**Figure 7:**
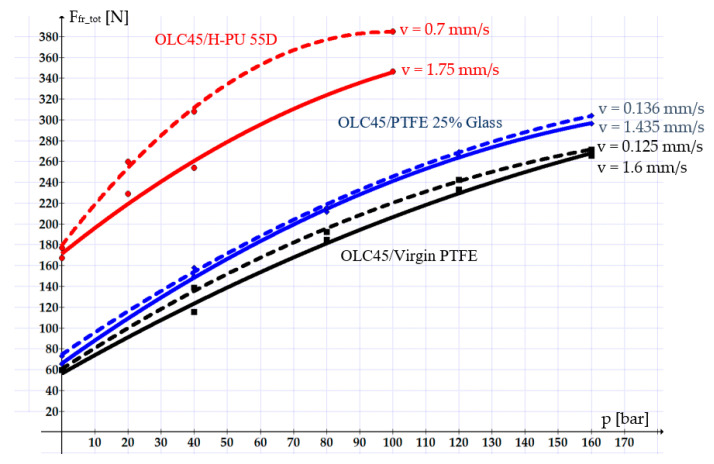
Variation in the total friction forces versus the velocity and the pressure of the sealed-off fluid.

Figure 7 shows the variation curves of the total friction forces for the three types of studied sealing systems. The dashed lines correspond to the smaller velocities, and the continuous lines correspond to the higher velocities.

The variation curves in Figure 7 (above) reveal that PTFE-based materials are preferable for manufacturing seals. The values of the friction forces obtained for seals made from Virgin PTFE or PTFE 25% Glass are 2 to 3 times smaller than the values measured for seals made from H-PU 55D. Between Virgin PTFE and PTFE 25% Glass, the utilization of the former is preferable, as, for this material, the friction forces are smaller by up to 20%.

**Figure 8:**
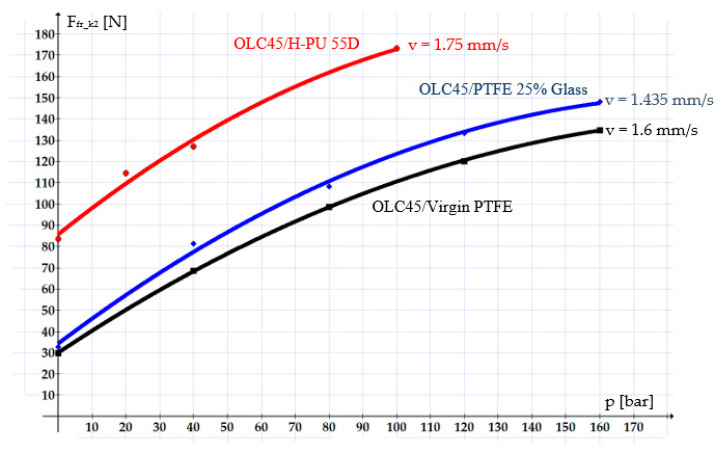
Variation in the friction forces in the case of a single coaxial sealing system.

Figure 8 shows curves describing the evolution of the individual friction forces of each sealing system. Of interest are the *F_fr_k_*_2_-type friction forces, as they describe the real behavior of a coaxial sealing system. The plotted curves refer only to the maximum tested velocities for each of the materials.

In all studied cases, the velocity stands out as a crucial influencing parameter of the friction forces: a higher velocity causes a smaller friction force. This is due to the fact that with increasing velocity, the thickness of the fluid layer between the components of the tribosystem also increases, leading to hydrodynamic friction. In cases involving small velocities, the fluid layer becomes thinner, and mixed-type or even dry friction sets in, causing increasingly greater friction forces.

The graphs in the figures above also show that the smallest friction forces occurred in the OLC45/Virgin PTFE pair, followed by OLC45/PTFE 25% Glass and OLC45/H-PU 55D. This can be explained mainly by the fact that Virgin PTFE and PTFE 25% Glass have smaller static friction coefficients than H-PU 55D (for example, for a working pressure of 100 bar and a velocity of 1.6 mm/s, the friction coefficients are as follows: μ_k Virgin PTFE_ = 0.021, μ_k PTFE 25% Glass_ = 0.0291, μ_k H-PU 55D_ = 0.0759).

This can also be explained by the sizeable fluor atoms with negative charges in PTFE that cancel out the positive charge carried by carbon atoms and thus enable the easy relative movement of the chains of molecules. Further, the low friction relayed by PTFE can be attributed to its stratified structure, not unlike that of compact lubricants [16].

A further parameter that influences the magnitude of the friction forces is the pressure of the working fluid. A higher working fluid pressure increases the friction forces. This can be explained by the fact that as the pressure on the sealing ring grows, the thickness of the fluid layer decreases. This allows the tips of the asperities of the two surfaces to cling one to another, thus hindering the relative motion.

Based on the results derived from this study, we recommend Virgin PTFE as the optimum material for sealing rings, followed by PTFE 25% Glass. In the case of small velocities, these two materials ensure the generation of small friction forces and smooth, uniform piston movement.

## 4. Conclusions

Reducing the friction forces generated by hydraulic cylinder sealing systems crucially contributes to improving the energy efficiency of hydraulic drives. Achieving the optimal selection of materials for sealing systems is a solution that ensures small friction forces. This paper analyzed the behavior of three polymer materials (Virgin PTFE, PTFE 25% Glass, and H-PU 55D) used for the sealing rings of hydraulic cylinder pistons.

First, a methodology for the calculation of the friction forces generated by hydraulic cylinder sealing systems was put forward. Subsequently, the experimentally determined values of the friction forces were presented and discussed, followed by recommendations concerning the choice of seal materials for hydraulic cylinders.

The results of this study led to a number of seminal conclusions:

- Compared to H-PU 55D, the friction is lower when Virgin PTFE and PTFE 25% Glass are used. Between Virgin PTFE and PTFE 25% Glass, the former performs better.
- For all the studied materials, if the pressure in the cylinder grows, the ensuing friction is greater.
- Any slowing down of the piston causes higher friction.

For the studied working conditions (pressure, velocity, material, roughness), the authors of the present study recommend the utilization of PTFE-type polymers. As the three studied materials are the most frequently used materials for hydraulic cylinder sealing systems, the conclusions reported in this study hold significant relevance for industry.

## Figures and Tables

**Table 1:** Relevant characteristics of the sealing ring materials.

Material	Composition	Sh D Hardness	Utilization
Virgin PTFE	100% PTFE	55 ± 3	Resistant to almost all chemicals
PTFE 25% Glass	25% clean milled glass fibers and 75% Virgin PTFE	58 ± 3	Resistant to almost all chemicals
H-PU 55D	PUR	55 ± 3	Resistant to oil, petrol, hot water, hot air, ozone

**Table 2:** Non-dimensional contact surface area *β*.

Pressure p [bar]							
0	20	40	60	80	100	120	160
Non-dimensional contact surface area *β* for Virgin PTFE							
0.025	0.092	0.159	0.225	0.292	0.359	0.425	0.559
Non-dimensional contact surface area *β* for PTFE 25% Glass							
0.043	0.158	0.272	0.386	0.50	0.615	0.729	0.958
Non-dimensional contact surface area *β* for H-PU 55D							
0.023	0.082	0.142	0.202	0.261	0.321	0.381	0.50

**Table 3:** Friction forces generated by the sealing systems for the OLC45/Virgin PTFE pair.

Pressure [bar]	*F_fr_tot_* [N]	Standard Deviation	*F_fr_k_*_1_ [N]	*F_fr_k_*_2_ [N]
v = 1.6 mm/s				
0	59.63	0.15165	29.81	29.81
40	115.4	0.18708	46.92	68.48
80	184.66	0.15165	85.98	98.68
120	232.74	0.18165	112.66	120.08
160	265.44	0.15165	130.69	134.75
v = 0.125 mm/s				
0	59.63	0.13038	29.81	29.81
40	138.49	0.22803	58.29	80.2
80	192.35	0.18165	89.78	102.57
120	242.36	0.18165	117.45	124.91
160	271.21	0.19235	133.57	137.64

**Table 4:** Friction forces generated by the sealing systems for the OLC45/PTFE 25% Glass pair.

Pressure [bar]	*F_fr_tot_* [N]	Standard Deviation	*F_fr_k_*_1_ [N]	*F_fr_k_*_2_ [N]
v = 1.435 mm/s				
0	65.4	0.17320	32.7	32.7
40	150.03	0.11402	68.74	81.29
80	211.58	0.10954	103.36	108.22
120	265.44	0.23021	132.1	133.34
160	296.22	0.13038	148.11	148.11
v = 0.136 mm/s				
0	73.09	0.08366	36.54	36.54
40	157.73	0.11401	72.5	85.23
80	215.43	0.25884	105.27	110.16
120	269.29	0.23874	134.02	135.27
160	303.91	0.14142	151.95	151.96

**Table 5:** Friction forces generated by the sealing systems for the OLC45/H-PU 55D pair.

Pressure [bar]	*F_fr_tot_* [N]	Standard Deviation	*F_fr_k_med_* [N]
v = 1.75 mm/s			
0	167.34	0.15165	83.67
20	228.9	0.15811	114.45
40	253.9	0.18708	126.95
100	346.23	0.13416	173.12
v = 0.7 mm/s			
0	176.96	0.18165	88.48
20	259.67	0.25884	129.83
40	307.76	0.16733	153.88
100	384.7	0.18708	192.35

## Data Availability

Data are contained within the article.
